# Comparison of the third-generation streamlined liner of the pharynx airway (SLIPA-3G) with the laryngeal mask airway supreme for laparoscopic cholecystectomy: a randomized prospective study

**DOI:** 10.1186/s12871-022-01638-0

**Published:** 2022-04-05

**Authors:** Hongna Fan, Lin Li, Lei Zhu, Zhuo Yi, Yugang Diao

**Affiliations:** General Hospital of Northern Theater Command, No. 83 Wenhua Road, Liaoning Shenyang, China

**Keywords:** SLIPA-3G, LMA Supreme, Supraglottic airway devices, Oropharyngeal leak pressure, Laparoscopic cholecystectomy

## Abstract

**Background:**

The third-generation streamlined liner of the pharynx airway (SLIPA-3G) is a new-generation supraglottic airway device (SAD) that is non-cuffed and disposable, with a sealing pressure that varies dynamically with the airway pressure. This study compared the SLIPA-3G with the laryngeal mask airway supreme (LMAS) in patients undergoing laparoscopic cholecystectomy.

**Methods:**

Two hundred and twenty patients scheduled for laparoscopic cholecystectomy were randomly allocated to either the SLIPA-3G group or the LMAS group. Data were collected on the patients’ hemodynamic parameters at different time points, ease of insertion, fiberoptic view, oropharyngeal leak pressure (OLP) at different time points and SAD-related complications.

**Results:**

The mean OLP immediately after device placement in the LMAS group was significantly higher than that of the SLIPA-3G group (31.34 ± 6.99 cmH_2_O vs.28.94 ± 6.01 cmH_2_O, *P* = 0.008, 95% CI 0.62–4.17). The OLPs of the two groups were not significantly different after the induction of a pneumoperitoneum until the end of surgery. The OLP increased gradually through the course of the operation in the SLIPA-3G group (*P* value = 0.035) but not in the LMAS group (*P* value = 0.945). There was no significant difference between the two groups in hemodynamic parameters, insertion time and success rate, fiberoptic view and complication rate.

**Conclusions:**

The SLIPA-3G and LMAS were associated with comparable OLPs, hemodynamic parameters, ease of insertion, fiberoptic views and complication rates when used during laparoscopic cholecystectomy. The SLIPA-3G can be used as an effective alternative to the LMAS in patients undergoing laparoscopic surgeries.

## Background

Supraglottic airway devices (SADs) have been widely used as minimally invasive alternatives to endotracheal tubes (ETTs). SADs possess a number of advantages over ETTs, including ease of insertion, minimal tracheal injury, hemodynamic stability and fewer complications such as sore throat [1]. There were initial concerns over the risk of aspiration associated with the use of SADs during laparoscopic surgeries, as the rise in intra-abdominal pressure may cause difficulties with ventilation and an increased risk of gastric regurgitation [2]. Numerous studies have demonstrated that second-generation SADs with esophageal drainage tubes can be safely used in patients undergoing elective laparoscopic surgeries without an elevated risk of aspiration [3–7].

The OLP is an important indicator of the efficacy of positive-pressure ventilation and airway protection for an SAD [8]. A meta-analysis looking at studies that compared the SLIPA with other conventional laryngeal mask airways (LMAs; Proseal LMA, classic LMA and SoftSeal LMA) found no differences between the two groups in terms of ease of insertion, oropharyngeal leak pressure (OLP) and quality of the fiberoptic view of the larynx [9].

**Fig. 1:**
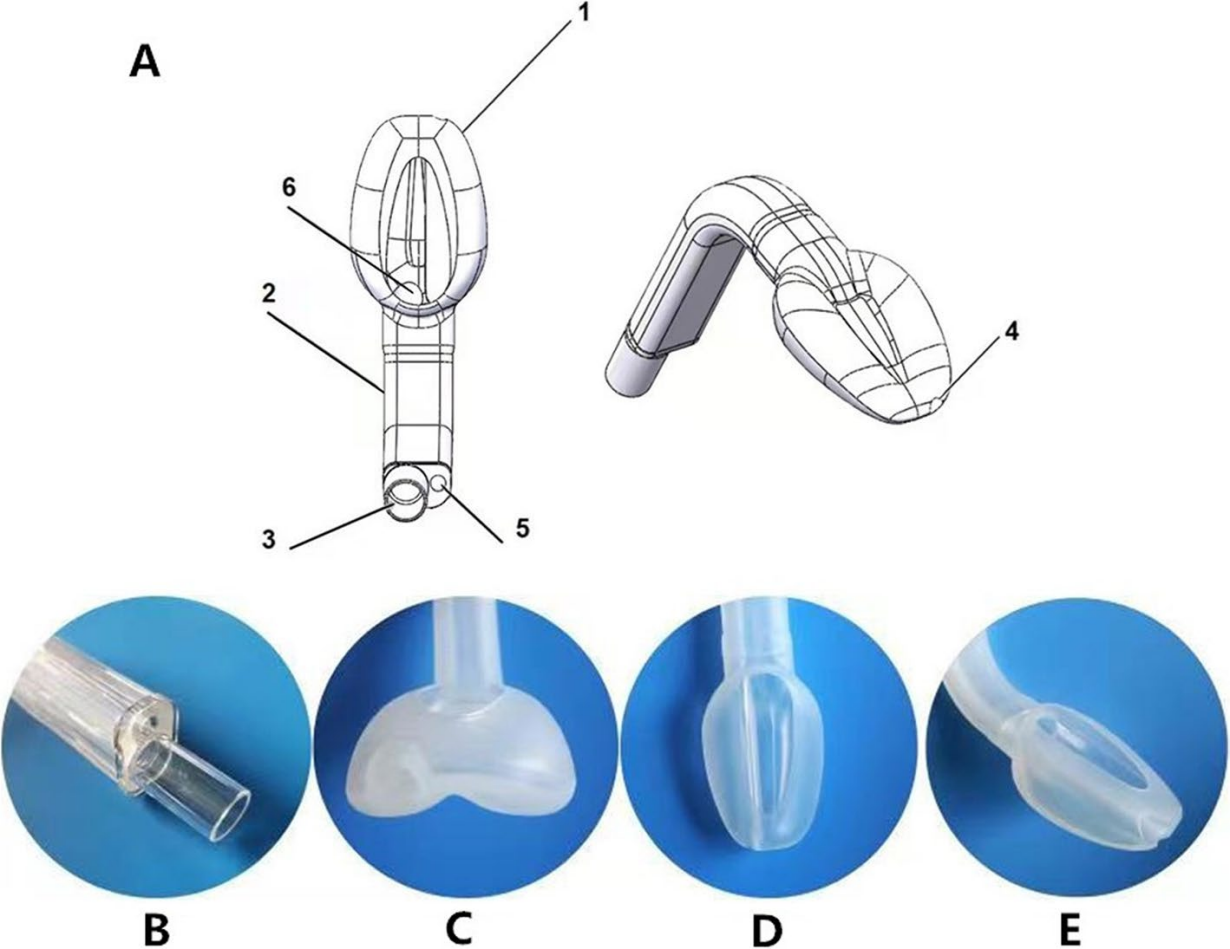
Design and features of the SLIPA-3G. **A** Illustration of features of the SLIPA-3G. 1, Self-sealing silicone cuff. 2, Silicone airway tube. 3, Airway connector. 4, Gastric tube outlet port. 5, Gastric tube inlet port. 6, Ventilating aperture. **B**-**E** Views of different parts of the SLIPA-3G

The third-generation SLIPA (SLIPA-3G) is the latest-generation SAD with a sealing pressure that varies dynamically with the airway pressure, i.e. a ‘self-energizing sealing device’ according to the Miller classification system [10]. It has a non-inflatable silicone cuff that is designed to conform to the human pharynx [11], creating a close perilaryngeal seal. The cuff has a thick and firm base and a thin tip. During positive pressure ventilation, the airway pressure equilibrates with the cuff pressure, resulting in tight oropharyngeal sealing. The new features in the SLIPA-3G include an additional passage for the gastric tube to allow for gastric decompression as well as an anti-reflux window on the posterior surface of the device to allow for rapid evacuation of regurgitated material from the oral cavity (Fig. 1). These unique features may provide additional protection against aspiration and airway obstruction due to regurgitated gastric content. In addition, it is manufactured with an in-built bite block and a reinforced shaft that allows for fiberoptic examinations. This is one of the first studies to evaluate the clinical application of the SLIPA-3G in laparoscopic surgery by comparing its OLP and other clinical parameters with those for the LMAS.

## Methods

### Study setting and population

 The study was approved by the PLA Northern Theater Command General Hospital Ethics Committee. All methods were carried out in accordance with relevant guidelines and regulations. Written informed consent was obtained from all participants. A total of 220 adults (American Society of Anesthesiologists physical status I-III) aged 18–70 years who were scheduled for elective laparoscopic cholecystectomy under general anesthesia were enrolled from August 2020 to December 2020. The exclusion criteria were (1) body mass index (BMI) > 30 kg/m^2^; (2) inadequate fasting (< 8 h); (3) suspected difficult airway; (4) oropharyngeal infection or other pathology; (5) latex allergy; and (6) emergency surgery.

### Study protocol

The participants were randomly allocated into one of two equal groups, the SLIPA-3G group and the LMAS group, based on numbers generated from a random number table. The random numbers were kept in sealed envelopes that were opened in the operating room by the anesthesiologist, just before the induction of general anesthesia. Both the investigators and the patients were blinded to the randomization and allocation.

Standard monitoring (pulse oximetry, electrocardiography, non-invasive blood pressure) and bispectral index (BIS) monitoring were applied in the operating room. All patients were premedicated with dexmedetomidine infusion at 1 µg/kg/hr for 10 min. Following preoxygenation with 100% oxygen for 3 min, anesthesia was induced with sufentanil 0.4–0.6 µg/kg, propofol 1–2 mg/kg and rocuronium 0.6 mg/kg. After loss of the lash reflex and a BIS value less than 50, the lubricated SLIPA-3G or LMAS was inserted and mechanical ventilation was initiated. In the SLIPA-3G group, the size of the device was selected by matching the width of the patient’s thyroid cartilage with that of the SLIPA-3G [12]. The anesthesiologist tilted the patient’s head using the left hand while inserting the toe of the SLIPA-3G towards the posterior pharynx using the right hand. With firm pressure applied on the chamber against the patient’s hard palate, the device was pushed down at the junction of the stem and chamber until resistance was felt. The entire induction process was performed by a single attending anesthesiologist.

Effective ventilation was confirmed with the presence of bilateral chest expansion and a square capnography waveform. If effective ventilation could not be confirmed, the SAD position was adjusted by rotating the device or changing its depth of insertion, or a difference size was used. After three unsuccessful attempts at placing either SAD, endotracheal intubation was performed and failed SAD placement was documented. A fiberoptic assessment of the glottic view was performed after the confirmation of effective ventilation.

Anesthesia was maintained using a balanced technique with inhaled sevoflurane at an end-tidal concentration of 2–4% in 60% oxygen and air, combined with propofol infusion at 50–150 µg/kg/min and remifentanil infusion at 0.1–0.2 µg/kg/min. Additional rocuronium was given as necessary to maintain adequate surgical relaxation. All patients were mechanically ventilated using the volume-controlled mode, with a tidal volume of 6–8 ml/kg and respiratory rate of 10–12 breaths per minute to maintain an end-tidal carbon dioxide reading in the range of 35–45 mmHg. At the end of the operation, neuromuscular blockade was reversed with neostigmine 2.0 mg and atropine 1.0 mg. The SAD was removed when the patient was awake and able to obey commands.

### Data collection

The following data were recorded:

1. Patient demographics: age, gender, height, weight, BMI.
2. Hemodynamic parameters: mean arterial blood pressure (MAP), heart rate (HR), and BIS before anesthesia induction (T0), immediately after SAD insertion (T1), five minutes after induction of the pneumoperitoneum (T2), five minutes after cessation of the pneumoperitoneum (T3), when the patient regained consciousness (T4) and immediately after SAD removal (T5).
3. Ease of insertion: success rate of first insertion, number of attempts, time taken for adequate placement (from jaw opening to effective supraglottic ventilation).
4. Fiberoptic view: The best views assessed from the tip of the orifice of the SLIPA-3G or LMAS were scored from 1 to 4 as described by Brimacombe and Berry as follows: 4, only vocal cords seen; 3, vocal cords and posterior epiglottis seen; 2, vocal cords and anterior epiglottis seen; 1, vocal cords not seen [13].
5. OLP – primary outcome: The OLP was measured using the “audible noise” method that was first described by Keller et al. [8]. When the LMAS was used, the cuff was inflated to a pressure of 60 cmH_2_O using a cuff manometer. With the adjustable pressure limiting valve of the anesthetic circuit closed at 70 cmH_2_O (manual ventilation mode) and a fresh gas flow rate of 3 L/min, the pressure at which an air leak was heard over the patient’s mouth was measured using (1) a calibrated aneroid cuff manometer attached to the proximal end of the SAD via a connecting tube and (2) the ventilator (i.e. airway pressure displayed on the anesthetic machine). The average of the two values measured with (1) and (2) was recorded as the OLP. The OLP was measured during T1, T2, T3 and T4.
6. Post-removal complications: The SAD was examined for the presence of blood stains once removed. The participants were interviewed by an independent, blinded investigator on the following day about any SAD-related adverse effects including sore throat and dysphagia.

### Statistical analyses

Sample size was calculated based on a pilot study conducted at our center, which found that the OLP of the LMAS was 28.1 ± 6.3 cmH_2_O and that of the SLIPA-3G was 31.1 ± 6.6 cmH_2_O. In order to detect an inter-device OLP difference of 3 cmH_2_O with a power of 0.9 and a type I error of 0.05, a sample size of 99 subjects per group would be required. A total of 110 patients were enrolled in each group to allow for potential dropouts. The Statistical Package for Social Sciences (SPSS) software (version 26 for Windows, SPSS Inc., Chicago, IL, USA) was used for all statistical analyses. Data were expressed as mean ± standard deviation, unless stated otherwise. Unpaired *t*-test was used to compare numerical data. Categorical variables were compared using the Chi-squared or Fisher’s exact test. Statistical significance was defined as a *P* value < 0.05.

## Results

### Sample

We recruited 220 patients for the study, among which six in the SLIPA-3G group and three in the LMAS group had endotracheal intubation performed after three unsuccessful attempts to place the SAD. These patients were excluded in the final analyses. There were no significant differences between groups in demographic data (Table 1).

**Table 1:** Demographic, anesthetic, and surgical data

	SLIPA-3G group	LMAS group	*P* value
Age (years)	52.1 ± 12.5	51.8 ± 12.9	0.855
Gender (male : female ratio)	0.63 ± 0.48	0.53 ± 0.50	0.135
Height (cm)	165.88 ± 7.44	166.82 ± 7.62	0.367
Weight (kg)	67.64 ± 10.44	68.99 ± 11.59	0.377
BMI	24.45 ± 2.81	24.71 ± 3.24	0.539
Duration of ventilation via SAD (min)	87.64 ± 25.86	91.73 ± 26.48	0.258
Duration of pneumoperitoneum (min)	38.20 ± 19.47	40.07 ± 22.10	0.517

Data are presented as mean ± standard deviation

### Primary outcome

Immediately after insertion, the mean OLP of the LMAS was significantly higher than that of the SLIPA-3G by 2.4 cmH_2_O (*P* value = 0.008). However, there was no significant difference in OLP between the two groups throughout the rest of the surgery (Table 2). Within-group analysis revealed that the OLP of the SLIPA-3G increased significantly as the surgery progressed from T1 to T4 (*P* value = 0.035). Such variation in OLP was not observed in the LMAS group (*P* value = 0.945).

**Table 2:** Oropharyngeal leak pressure (cmH_2_O)

	SLIPA-3G group	LMAS group	*P* value
T1	28.94 ± 6.01	31.34 ± 6.99	0.008
T2	29.97 ± 6.89	30.74 ± 7.71	0.445
T3	31.19 ± 7.06	30.88 ± 7.64	0.757
T4	31.37 ± 7.18	30.93 ± 7.57	0.662

Data are presented as mean ± standard deviation. *T1 *immediately after SAD insertion, *T2 *five minutes after induction of pneumoperitoneum, *T3 *five minutes after cessation of pneumoperitoneum, *T4 *when patient regained consciousness

### Secondary outcomes

There were no significant differences between the SLIPA-3G and LMAS with regard to the patients’ hemodynamic parameters (Table 3), ease of insertion and fiberoptic views (Table 4). Placement of the SLIPA-3G was successful on the first attempt 67.3% of the time, compared to 71.0% for insertion of the LMAS. It took an average of 18.83 s and 16.46 s respectively to properly position a SLIPA-3G or LMAS.

**Table 3:** Hemodynamic parameters

	SLIPA-3G group	LMAS group	*P* value
MAP			
T0	102.75 ± 15.57	103.26 ± 13.86	0.801
T1	86.58 ± 16.65	88.39 ± 15.70	0.416
T2	88.09 ± 14.44	89.27 ± 11.95	0.517
T3	83.39 ± 9.62	83.86 ± 12.62	0.764
T4	91.93 ± 15.12	95.19 ± 15.94	3.25
T5	96.16 ± 12.94	97.74 ± 14.51	0.407
HR			
T0	78.77 ± 15.41	76.04 ± 13.46	0.252
T1	62.25 ± 13.52	65.56 ± 14.49	0.088
T2	55.63 ± 11.22	57.36 ± 9.23	0.225
T3	53.77 ± 9.36	54.04 ± 9.06	0.833
T4	66.21 ± 11.28	65.61 ± 12.71	0.716
T5	69.62 ± 10.60	67.76 ± 11.39	0.222
BIS			
T0	96.33 ± 1.32	96.52 ± 1.15	0.251
T1	42.62 ± 1.65	42.77 ± 2.16	0.570
T2	43.04 ± 1.64	43.44 ± 1.94	0.109
T3	48.01 ± 6.38	46.47 ± 5.08	0.053
T4	83.26 ± 3.51	82.89 ± 4.23	0.489
T5	87.62 ± 4.04	88.29 ± 3.99	0.224

Data are presented as mean ± standard deviation. *T0 *before anesthesia induction, *T1 *immediately after SAD insertion, *T2 *five minutes after induction of pneumoperitoneum, *T3 *five minutes after cessation of pneumoperitoneum, *T4* when patient regained consciousness, *T5 *immediately after SAD removal

**Table 4:** Insertion parameters

	SLIPA-3G group	LMAS group	*P* value
Number of insertion attempts			0.318
One attempt	70 (67.3%)	76 (71.0%)	
Two attempts	30 (28.8%)	28 (26.2%)	
Three attempts	4 (3.9%)	3 (2.8%)	
Overall success rate	104 (94.5%)	107 (97.3%)	
Insertion time (seconds)	18.83 ± 11.23	16.46 ± 11.68	0.135
Fiberoptic view 1/2/3/4	15/21/28/40	8/30/19/50	0.089

Data are presented as number of patients. Insertion time is defined as time taken from jaw opening to effective supraglottic ventilation

After device removal, blood stains were noted in 7.7% of cases in the SLIPA-3G group and in 7.5% of cases in the LMAS group. Thirteen patients in the SLIPA-3G group and 11 in the LMAS group complained of a sore throat. Two patients in the LMAS group had dysphagia, which was not experienced by any patient in the SLIPA-3G group. There was no significance difference in the overall complication rates between the groups (Table 5). Gastric aspiration was not observed in either group.

**Table 5:** Post-removal complications

	SLIPA-3G group	LMAS group	*P* value
Blood stains	8 (7.7%)	8 (7.5%)	
Sore throat	13 (12.5%)	11 (10.2%)	
Dysphagia	0	2 (1.0%)	
Total	21 (20.2%)	21 (19.6%)	0.163

Data are presented as number of patients

## Discussion

Owing to its unique design, the SLIPA has a few potential advantages over conventional SADs. The device conforms to the hypopharynx, negating the need for an inflatable cuff. Without a sealed cuff, the interior of the bowl of the SLIPA is in continuous with the patient’s airway. As a result, the pressure exerted by the device on the upper airway mucosa equilibrates with the patient’s airway pressure. Based on the same principle, the SLIPA’s OLP may vary with the patient’s airway pressure. This was indeed observed in our study where the SLIPA-3G’s OLP increased gradually after induction of the pneumoperitoneum. Although the LMAS’s group had higher OLP immediately after insertion, there was no significant variation in the OLP throughout the course of the surgery. Pneumoperitoneum decreases chest wall compliance through the lower side. There is, as a consequence, an increase in airway pressure. To attain a similar transpulmonary pressure, this “new” (higher) pressure therefore fills the SLIPA cuff with extra volume, resulting in a stronger seal [14]. Another proposed explanation for the rise in SLIPA’s OLP over-time is the softening of the device at body temperature, thereby producing a more effective perilaryngeal seal [15]. Such dynamic variation in the SLIPA’s OLP may provide unique benefits in situations where the patient’s airway pressure may increase intraoperatively e.g. during laparoscopic surgery.

SAD insertion is known to cause less hemodynamic disturbances compared to endotracheal intubation [16]. In this study, we found no significant difference in the patients’ hemodynamic parameters at different time points between the two groups. We also observed a general drop in the patients’ MAP, HR and BIS values after induction and placement of SAD in both groups. This study’s results agree with previous study results that the SLIPA-3G, like the LMAS, does not cause significant hemodynamic responses during insertion.

The SLIPA is designed based on the human pharyngeal anatomy [11]. Combined with a dynamically equilibrating pressure between the device and the upper airway mucosa, which is unique to the SLIPA-3G, upper airway injuries caused by over-inflation of SAD cuffs such as sore throat [17] and dysphagia may potentially be reduced. In this study, we observed similar complication rates between the SLIPA-3G and LMAS groups. However, our study has insufficient power to detect any difference in postoperative complications. Further studies will be required to evaluate the safety of the device. The device is smaller than LMAS, potentially facilitating easy insertion. Our study showed that the SLIPA-3G had a similar insertion time, success rate and fiberoptic views as the LMAS in the hands of experienced anesthesiologists. Previous studies have found that the SLIPA is easier to insert for novices compared to other conventional SADs [18–20]. The SLIPA may therefore be useful as a rescue airway in resuscitation scenarios, where the operator may not be an anesthesiologist or have much experience with the insertion of SADs.

## Conclusions

In our study, we found that the SLIPA-3G, in comparison to the LMAS, provide for a similar OLP after induction of the pneumoperitoneum and stable patient hemodynamic parameters during laparoscopic cholecystectomy. The SLIPA-3G could be easily inserted with an overall success rate of 94.5% after three attempts. It was associated with similar fiberoptic views and complication rates as the LMAS. Therefore, the SLIPA-3G is an effective SAD that can be used in mechanically ventilated patients undergoing laparoscopic surgeries.

## Data Availability

The datasets used and/or analysed during the current study are available from the corresponding author on reasonable request.

## Abbreviations

SLIPA-3G: Streamlined Pharynx Airway Liner Airway-3 g
LMAS: Laryngeal Mask Airway Supreme
OLP: Oropharyngeal Leakage Pressure
SAD: Supraglottic Airway Device
ETT: Endotracheal tube
BMI: Body Mass Index
MAP: Mean Arterial Pressure
HR: Heart Rate
BIS: Bispectral Index
